# Recent advances on development of p21-activated kinase 4 inhibitors as anti-tumor agents

**DOI:** 10.3389/fphar.2022.956220

**Published:** 2022-08-29

**Authors:** Yang Li, Qing Lu, Chenghu Xie, Yiming Yu, Ao Zhang

**Affiliations:** ^1^ Pharm-X Center, School of Pharmacy, Shanghai Jiao Tong University, Shanghai, China; ^2^ School of Pharmaceutical Sciences, Wenzhou Medical University, Wenzhou, China

**Keywords:** p21-activated kinase 4, oncotherapy, immune infiltration, small molecular inhibitors, clinical progress

## Abstract

The p21-activated kinase 4 (PAK4) is a member of the PAKs family. It is overexpressed in multiple tumor tissues. Pharmacological inhibition of PAK4 attenuates proliferation, migration, and invasion of cancer cells. Recent studies revealed that inhibition of PAK4 sensitizes immunotherapy which has been extensively exploited as a new strategy to treat cancer. In the past few years, a large number of PAK4 inhibitors have been reported. Of note, the allosteric inhibitor KPT-9274 has been tested in phase Ⅰ clinic trials. Herein, we provide an update on recent research progress on the PAK4 mediated signaling pathway and highlight the development of the PAK4 small molecular inhibitors in recent 5 years. Meanwhile, challenges, limitations, and future developmental directions will be discussed as well.

## 1 Introduction

p21-activated kinases (PAKs), a family of serine/threonine kinases, were first reported as downstream signaling molecules of Cdc42 and Rac1 (Manser et al., 1994). Accumulating evidence have disclosed that PAKs are cross-linked with multiple signaling pathways, including WNT, ErbB2, MAPK, and many others (Radu et al., 2014; Kumar et al., 2017). Furthermore, PAKs also play crucial roles in cytoskeleton remodeling, cell adhesion, mitosis, neural differentiation, and cardiomyogenesis (Kelly and Chernoff, 2012; Kuzelova et al., 2021; Zhang et al., 2022). Recently, PAKs were found overexpressed in various cancers, and closely implicated in cancer-related signaling pathways (Rane and Minden, 2019).

Due to the homology of protein structure and sequence, PAKs are divided into group Ⅰ (PAK1-3) and group Ⅱ (PAK4-6) (Rudolph et al., 2015). All isoforms of PAKs contain the Cdc42/Rac1 interaction domains, however, group Ⅰ PAKs are activated by either Cdc42 or Rac1, whereas activation of group Ⅱ PAKs is solely dependent on Cdc42 (Radu et al., 2014). Among the PAKs family, PAK3, PAK5, and PAK6 are mostly expressed in the neuronal tissues, while PAK1, PAK2, and PAK4 are ubiquitous (Kelly and Chernoff, 2012; Rudolph et al., 2015). Many preclinical and clinical studies have reported that overexpression or genomic amplification of PAK1, PAK2, and PAK4 is often detected in multiple tumor tissues, including pancreatic, ovarian, breast, and gastric cancers (King et al., 2014; Ong et al., 2015; Wang et al., 2015; Prudnikova and Chernoff, 2017; Wang et al., 2020), warranting these three isoforms as potential targets for cancer treatment. However, a recent study alarmed that inhibition of PAK1 or PAK2 or both induced acute cardiovascular toxicity (Rudolph et al., 2016), indicating that pan-PAKs inhibitors, especially pan-group Ⅰ PAKs inhibitors, may not benefit the patients in the clinic.

**FIGURE 1:**
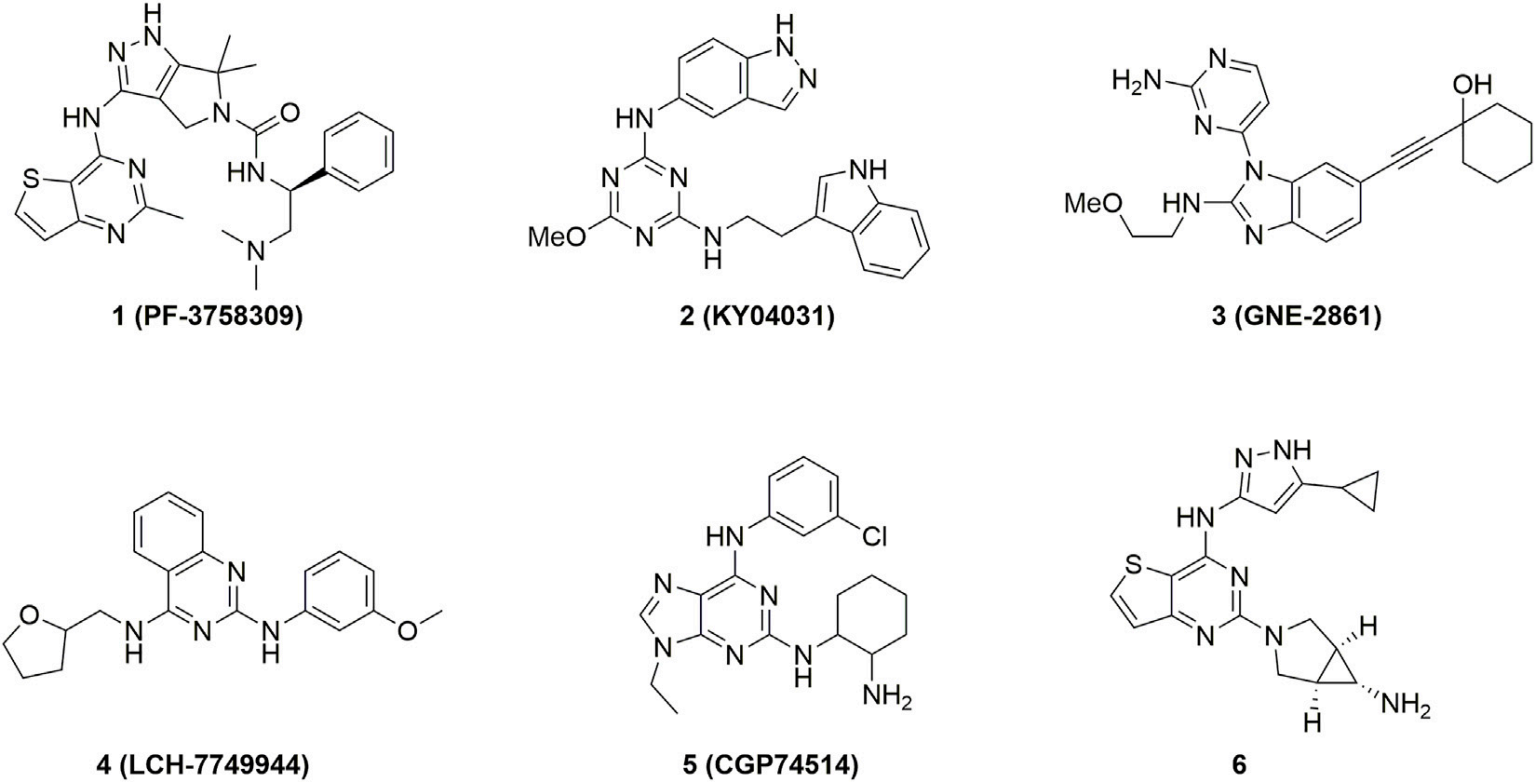
Representative PAK4 inhibitors developed before 2016.

Recently, more and more evidences suggested that PAK4 might be an ideal target for treat certain cancers (Santiago-Gómez et al., 2019; Wang F et al., 2019; Dasgupta et al., 2021). Inhibition of PAK4 was found to attenuate cancer cell migration and invasion, and suppress growth of diverse tumors *in vivo* (Guo et al., 2020; Fu et al., 2021). Furthermore, PAK4 was also found as an important regulator of tumor immunity (Nasmall Yi et al., 2021), thus opening a novel landscape for PAK4-related therapy. Since the latest review on the development of PAK4 inhibitors in 2015 (Figure 1) (Rudolph et al., 2015), tremendous progress have been achieved both in the discovery and development of new-generation inhibitors as well as the underlying biological mechanism. Hence, we review the most recent development of PAK4 inhibitors since 2016 with full accounts on the preclinical and clinical results both as monotherapy and in combination with immunotherapy.

## 2 The structure, the function and the expression of p21-activated kinase 4

**FIGURE 2:**
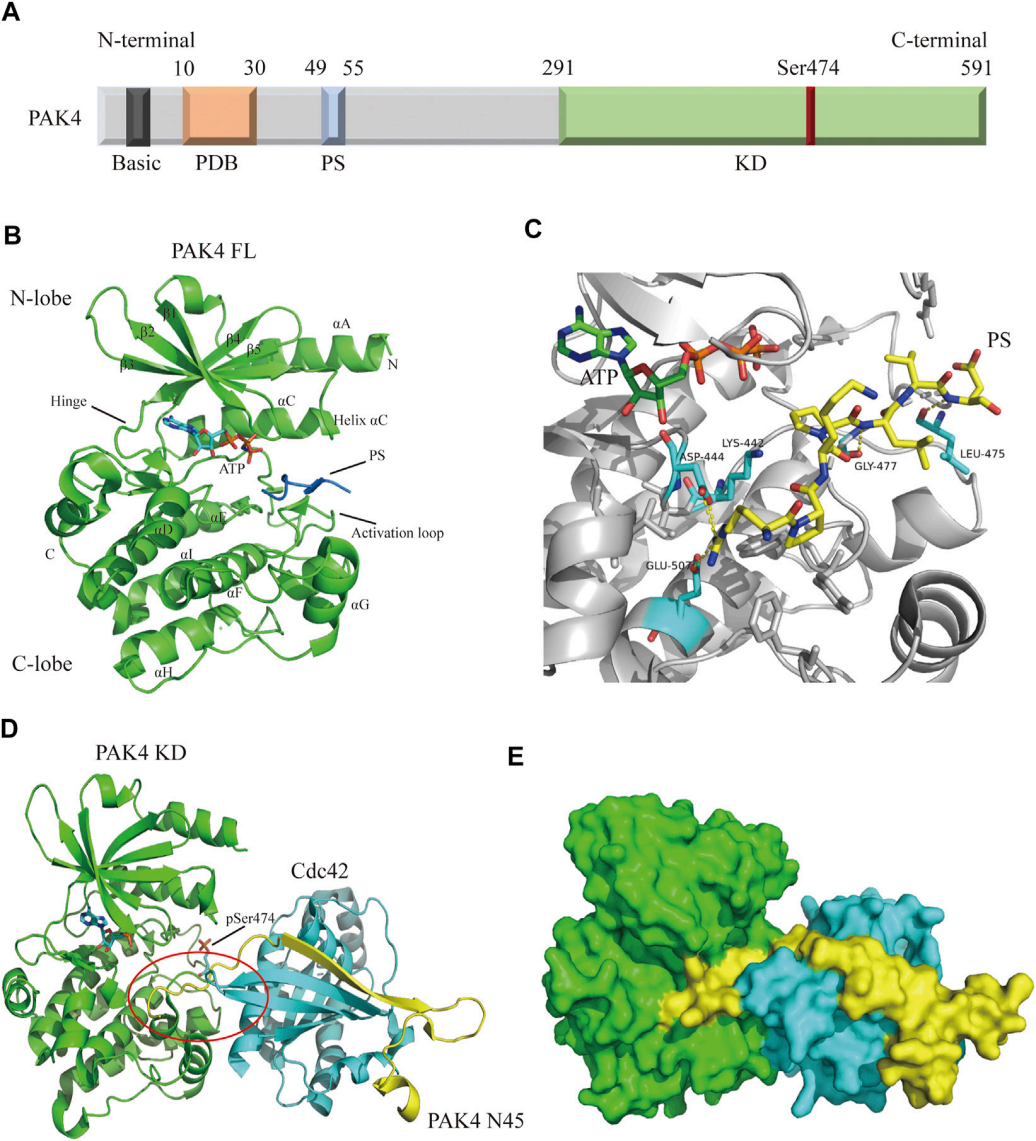
Overall structure of PAK4. **(A)** Schematic diagram of PAK4 domain structure. The regions are colored and labeled. Amino acid number of regions has shown. **(B)** Crystal structure of PAK4 FL (PDB code: 4FIE). The secondary structures have been labeled. PS is colored blue. **(C)** The detailed model of PS (yellow) binding to substrate binding site. Hydrogen bonds are colored yellow. **(D)** The ternary complex of PAK4 KD/PAK4 N45/Cdc42 (PDB code: 5UPK). PAK4 KD is colored green. PAK4 N45 is colored yellow. Cdc42 is color blue. **(E)** Surface diagram of the ternary complex which is shown in 2D. PDB: p21 binding domain, PS: Pseudosubstrate, KD: Kinase domain, FL: Full-length, N45: N terminus (residue 1-45).

Structurally, the PAK4 protein contains a C-terminal kinase domain (KD), an N-terminal p21-binding domain (PBD), a pseudosubstrate sequence (PS) or an autoinhibitory domain (AID), and a basic residue cluster for membrane targeting (Figure 2A) (Radu et al., 2014; Sampat and Minden, 2018). The precise mechanism of PAK4 activation remains unclear, but two interaction models have been proposed. In one model, (Baskaran et al., 2012) PAK4 is constitutively phosphorylated on Ser474 in the activation loop, but the activity is autoinhibited by AID through blocking substrate access, until the GTP-bound Cdc42 binds to PDB leading to a conformational activation in AID. In another model, (Ha et al., 2012) PAK4 contains a PS, which inserts in the substrate-binding site of the kinase domain to inactivate the protein (Figure 2B), however, when GTP-bound Cdc42 binds to PDB, the protein is relocated, and the SH3 section-containing protein binds with the PS to unlock the KD, leading to PAK4 activation. The crystal structure of full-length (FL) PAK4 suggests that the N-terminal PS tightly interacts with residues surrounding the substrate-binding pocket *via* abundant hydrogen bonds, ensuring a tight autoinhibition state (Figure 2C). Cdc42 was previously considered only to interact with the PBD of PAK4, however, a recent study reveals that Cdc42 contacts both PBD and KD of PAK4, thus forming a compact complex (Ha and Boggon, 2018). The binding of Cdc42 partially disturbs autoinhibition of PAK4 by PS, and forms a ternary complex with both the N-terminus and KD of PAK4 (Figures 2D,E). The N-terminus extends from the substrate-binding site of KD to the Cdc42 and connects them to form a complex, which induces a secondary inhibition in a pseudosubstrate-like manner (Ha and Boggon, 2018). Moreover, an endogenous inhibitor Inka1 was reported to block the activation of PAK4 by interacting with the substrate-binding pocket (Baskaran et al., 2015).

PAK4 is ubiquitously expressed in tissues and is of substantial importance in cytoskeletal organization, cell adhesion, and pathway regulation (Kelly and Chernoff, 2012). PAK4 also plays a key role in heart formation during embryonic development. The level of PAK4 was found highly expressed in the developing heart but decreased in the mature mouse heart (Nekrasova and Minden, 2012). Deletion of PAK4 in the progenitor caused aberrant upgrowth of the outflow tract, leading to the aortic wall thinner (Nekrasova and Minden, 2012). Knockout of the embryo PAK4 reduced expression of cytoskeletal organization related protein LIMK1 and induced abnormal cardiac development (Nekrasova and Minden, 2012), which may eventually lead to the embryo death. Furthermore, PAK4 was also found to mediate the neuronal development and neuroprotection (Zhang et al., 2022). The absence of neurodevelopment and a thinner neuroepithelium around the hindbrain and forebrain were observed in the PAK4-null embryos. Meanwhile, the defect of motor neuron differentiation was observed as well in such conditions (Qu et al., 2003). In hSOD1^G93A^ mice, a high level of PAK4 in the spinal neurons was found to block degeneration of motor neuron, thus prolonging survival rates (Cong et al., 2021).

**FIGURE 3:**
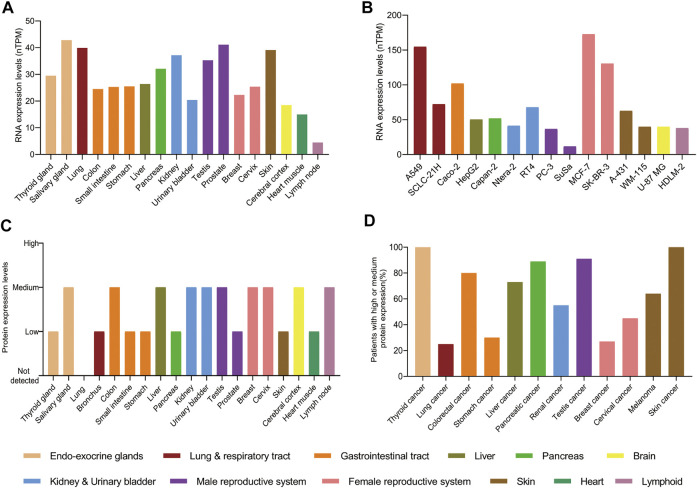
The protein and RNA levels of PAK4. **(A)** The RNA levels of PAK4 in normal tissues. **(B)** The RNA levels of PAK4 in different cancer cell lines, nTPM: normalized transcript per million value. **(C)** The protein levels of PAK4 in normal tissues. **(D)** The percentage of patients with high or medium protein levels of PAK4 in various tumors. The tissues are distinguished in different colors. The data were integrated from www.proteinatlas.org.

In adults, the levels of RNA and protein of PAK4 are detected in almost all tissues with no obvious specificity, although the level of RNA expression is generally low and the protein expression is within low to medium (Figures 3A,C). However, the levels of RNA and protein are elevated in cancer cells and tissues. For example, the levels of RNA are significantly increased in cancer cell lines, which derived from respiratory tract, gastrointestinal tract and female reproductive system. Particularly, a 6 to 8-fold RNA increase is observed in the SK-BR-3 and MCF-7 cell lines compared with the normal breast tissue. Similarly, there is a 3-fold increase of RNA level in the A549 cell line compared with the lung (Figure 3B). Moreover, the protein of PAK4 is hardly detectable in the lung, but medium to high levels were found in nearly 20% of patients suffering from lung cancer. Similar increase of PAK4 protein is observed in thyroid, stomach, pancreatic, and skin cancers as well. Furthermore, over 80% of patients bearing thyroid, colorectal, pancreatic, testis, and skin tumors have medium to high expression of PAK4 protein. Especially, all patients suffering from thyroid cancer or skin cancer have medium to high PAK4 expression (Figure 3D). Taken together, PAK4 is widely distributed in normal human tissues with low levels, and overexpressed in tumors, a clear indication of correlation between PAK4 and cancer.

## 3 The role of p21-activated kinase 4 in cancer

### 3.1 P21-activated kinase 4 mediated cancer through multiple signaling pathways

**FIGURE 4:**
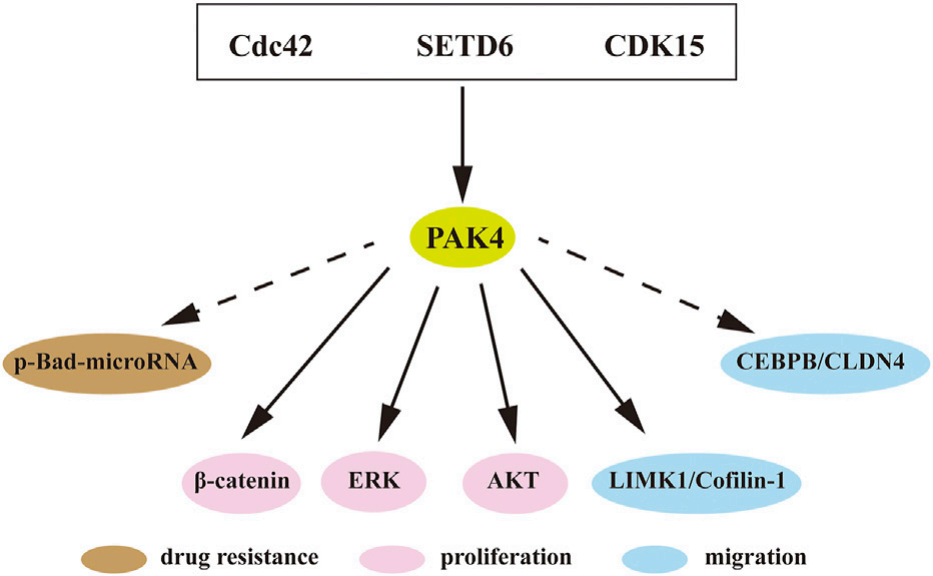
PAK4 mediated signaling pathways. Direct or indirect interactions of PAK4 with downstream regulators to control cell proliferation, migration and drug resistance. Cdc42, cell division control protein 42; SETD6, SET-domain containing protein 6; CDK15, cyclin-dependent kinase 15; Bad, Bcl-2-associated death promoter; MEK, mitogen-activated extracellular signal regulated kinase; AKT, protein kinase B; LIMK1, LIM domain kinase 1; CEBPB, CCAAT/enhancer-binding protein β; CLDN4, Claudin-4. Dotted arrow indicates indirect interaction, solid arrow indicates direct interaction.

A large number of studies have shown that PAK4 is intimately involved in proliferation, migration, invasion, drug resistance and immune escape of cancer cells (Ha et al., 2015; Won et al., 2019). Amplification of the PAK4 gene and dysregulated activation of the PAK4 protein are commonly observed in ovarian cancer, breast carcinoma, and pancreatic carcinoma (Ha et al., 2015; Won et al., 2019). The underlying mechanisms of PAK4 include WNT, Raf/MEK/ERK, PI3K/AKT, and many among others (Figure 4).

KRAS is located in upstream of PAK4 and often aberrantly activated in various tumors. The KRAS-driven HCT116 cell was found highly sensitive to PAK4 inhibition. Knockdown of PAK4 restrained the proliferation of KRAS-driven HCT116 cells *in vitro* (Tabusa et al., 2013). In rhabdomyosarcoma, inhibition of PAK4 blocked the RAS-mediated signaling pathway and suppressed tumor growth *in vivo* (Dasgupta et al., 2021). The WNT/β-catenin pathway is essential in cancer progression. The substrate β-catenin was found directly binding to PAK4 and is then phosphorylated on Ser675 to promote its intracellular translocation and signaling (Li et al., 2012). In addition, recent studies revealed that SET-domain containing protein 6 (SETD6) induces PAK4 methylation, which then suppresses cell adhesion by β-catenin activation (Vershinin et al., 2016; Vershinin et al., 2020). Furthermore, CDK15 is an upstream protein of PAK4 and functions as a regulator of PAK4 phosphorylation to elevate the activity of β-catenin and MEK/ERK signaling in colorectal cancer (Huang et al., 2022). It is known that LIM domain kinase 1 (LIMK1)-Cofilin pathway is involved in cytoskeletal formation (Dan et al., 2001). PAK4 interacts with LIMK1, and activates the LIMK1-Cofilin pathway to induce cytoskeletal change. Over-activation of this pathway was detected in osteosarcoma, which induces cell proliferation, invasion, and migration. PAK4 inhibition can attenuate this pathway and block the progression of prostate cancer and osteosarcoma (Zhang et al., 2019; Li R et al., 2020). Suppression of PAK4 was also found to mitigate activation of the PI3K/AKT pathway and inhibited progression of breast cancer (He et al., 2016).

In addition to the classical signaling pathways, PAK4-related axes were also reported associated with cancer. *p*-Bad-microRNA axis regulates drug resistance in pancreatic ductal adenocarcinoma (PDAC), and blocking of PAK4 induces reduction of Bad phosphorylation and upregulation of tumor-suppressive miRNAs in PDAC (Mohammad et al., 2019). The expression of Claudin-4 (CLDN4) is positively correlated with PAK4 in breast cancer and PAK4 induces CCAAT/enhancer binding protein *β* (CEBPB) activation, resulting in upregulation of CLDN4. Blocking of PAK4 decreased the activation of PAK4/CEBPB/CLDN4 and subsequently suppressed migration and invasion of breast cancer (Wang M et al., 2019).

### 3.2 P21-activated kinase 4 involved in T cell infiltration

Immune escape is one of the essential hallmarks of cancer (Hanahan, 2022). Aberrant vascularity surrounding tumor tissue constitutes the immune suppressive microenvironment that reduces immune cell infiltration and dampens the therapeutic effects of immunological treatment (Schaaf et al., 2018). Furthermore, multiple intrinsic oncogenic events of cancer cells have been found to promote immune escape (Spranger and Gajewski, 2018). Recent studies revealed that PAK4 plays an important role in promotion of immune escape of cancer cells, warranting for PAK4 as a promising target for immunotherapy.

**FIGURE 5:**
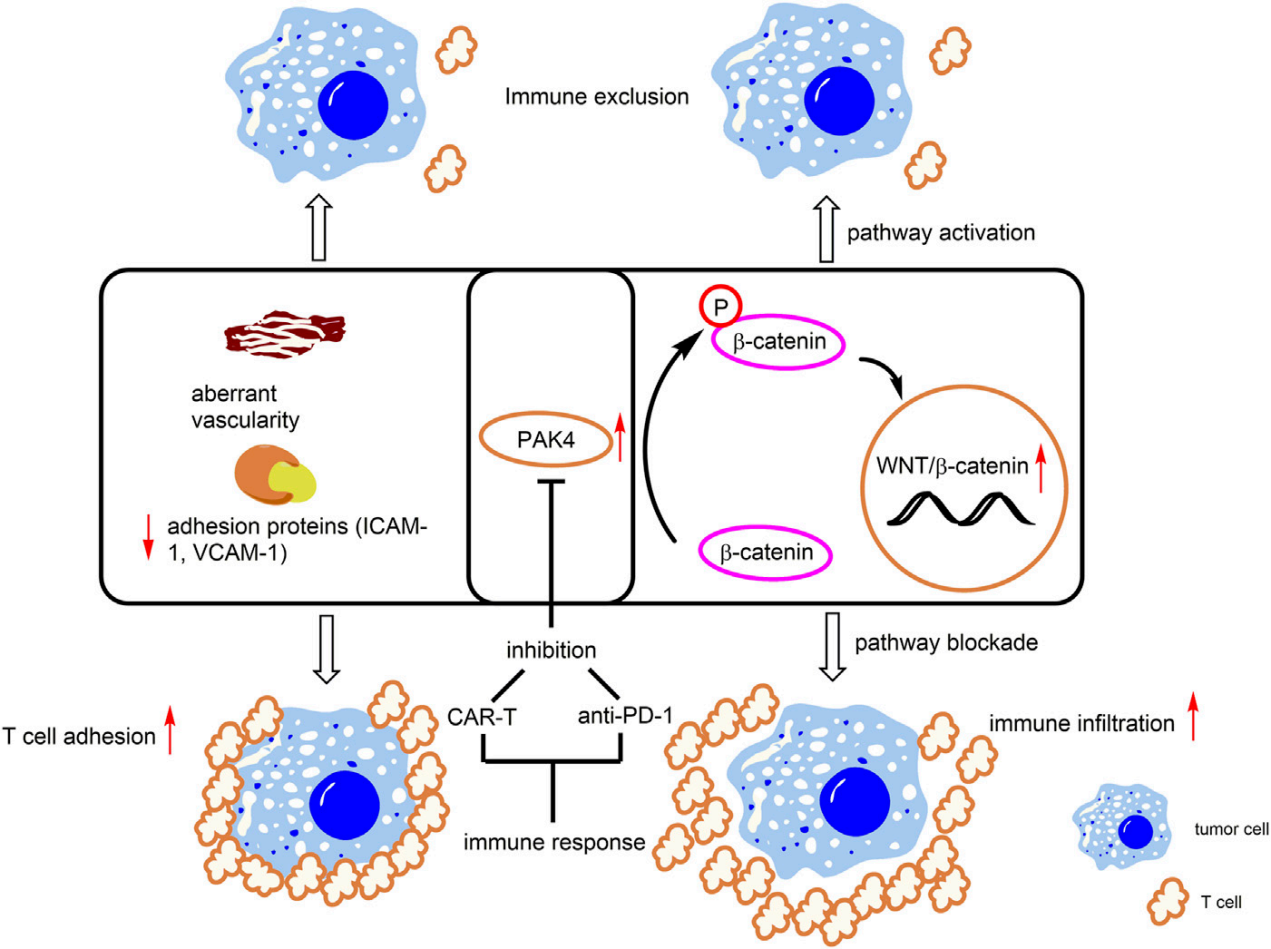
PAK4-mediated immune responses. Overexpression of PAK4 induces β-catenin phosphorylation and activates the WNT/β-catenin pathway. In addition, PAK4 decreases levels of T cell related adhesion protein (ICAM-1, VCAM-1) and induces aberrant vascularity. These events lead to immune exclusion. Knockdown or pharmacological inhibition of PAK4 promotes T cell infiltration and immunotherapeutic response.

PD-1/PD-L1 is the most successful immune checkpoint target of tumor immunotherapy and its blocking antibodies have been found to exert significant clinic therapeutic effects in various tumors, thus having been widely prescribed by tumor patients (Gajewski and Fessler, 2020). However, continuous treatment of PD-1/PD-L1 blockers also suffers from drug resistance, which is in large part due to lack of immune cells or poor T cell infiltration in surrounding tumor tissues. Notably, overexpression of PAK4 was detected in biopsies of tumor patients who lack immune cell infiltration and negative correlation between PAK4 expression and T cell infiltration has been evidenced in various tumors, such as pancreatic and prostate cancers featuring poor PD-1 blockade response (Abril-Rodriguez et al., 2020). In addition, several studies revealed that PAK4 and β-catenin are both elevated in tumors with low T cell infiltration (Abril-Rodriguez et al., 2020) (Figure 5). In the PAK4 knockout (KO) B16 cell, β-catenin phosphorylation was decreased and treatment with PD-1 blockade significantly reduced tumor growth (Abril-Rodriguez et al., 2020). All these results demonstrate that PAK4 is a promising immuno-therapeutic target with potential to overcome drug resistance of immune checkpoint inhibitors.

Through genetic screening, PAK4 is identified as a crucial regulator for proliferation, migration, invasion and permeability of glioblastoma-derived endothelial cells (GBM ECs) (Ma et al., 2021). In normal ECs, the basic functions are not affected with specific PAK4 KO, however, the animal survival rate and vascular abnormality are obviously improved after PAK4 KO in the GBM EC model. In addition, KO of PAK4 was found to promote T cell infiltration through enhancing the levels of adhesion proteins, including ICAM-1 and VCAM-1, to reinforce T cell adhesion to the vasculature of the GBM ECs (Ma et al., 2021) (Figure 5). Further, the combination of a PAK4 inhibitor with CAR-T therapy was found to improve mice survival rate, compared with monotherapy in the GBM model. (Ma et al., 2021). These results demonstrated that PAK4 is an important impact factor and a promising drug target for aberrant vascularity and T cell infiltration.

## 4 Recent advances on the development of p21-activated kinase 4 inhibitors

### 4.1 ATP competitive inhibitors

#### 4.1.1 Indolin-2-one based inhibitors

**FIGURE 6:**
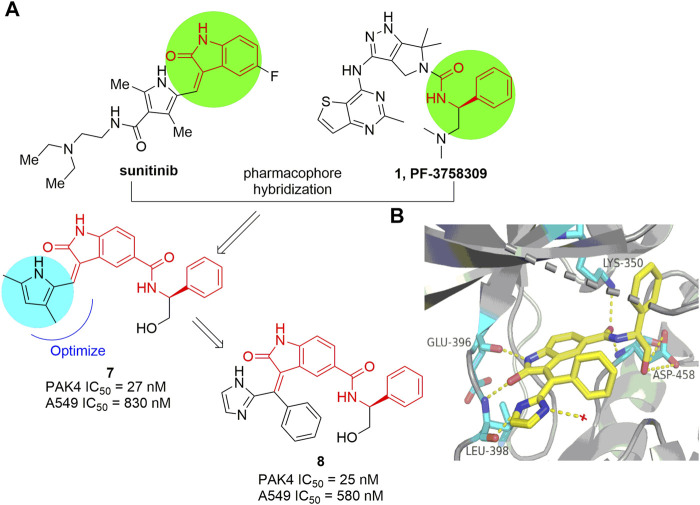
**(A)** The pharmacophore hybridization and subsequent optimization leading to compounds 7 and 8. **(B)** The crystal structure of compound 8 binding to PAK4 (PDB code: 5ZJW).

The indolin-2-one scaffold is a classical pharmacophore of protein kinase inhibitors. Through pharmacophore hybridization of the indolin-2-one motif of the multiple kinase inhibitor sunitinib and the N-acyl benzylamine motif of the early developed pan-PAK inhibitor PF-3758309 (Figure 1), a series of 5-acyl-indolin-2-one derivatives were designed (Guo et al., 2017). Among them, compound 7 (Figure 6A) inhibits PAK4 with IC_50_ values of 27 and 830 nm respectively in the biochemical and the proliferation assay against A549 cells. Mechanism study confirms that 7 inhibits phosphorylation of PAK4 and its downstream proteins LIMK1 and Cofilin, and mitigates cancer cell migration and invasion. Further optimization provided the analogue 8 (Figure 6A) with IC_50_ values of 25 nm against PAK4 and 580 nm against the growth of A549 cells, respectively (Guo et al., 2018). The crystal structure of 8 in complex with the ATP pocket of PAK4 reveals that the indolin-2-one motif interacts with the key amino acid residues Glu 396 and Leu 398 *via* hydrogen bonds (Guo et al., 2018). Meanwhile, multiple hydrogen bonds are formed between the carbonyl, NH, the terminal hydroxyl in the C5-position of the indolin-2-one component and the Lys 350 and Asp 458 as well (Figure 6B). In addition, the imidazole moiety forms a hydrogen bonding with a water molecule. Notably, the phenyl linked by alkene, inserts in a narrow lipophilic cleft with vertical conformation. All these interactions contribute to the elevated PAK4 potency of compound 8. Unfortunately, despite of the indolin-2-one core proving as a new scaffold for PAK4 inhibitor, compound 8 potently suppressed many off-targets as well, including BRAF, PDK1, CDK2, and others (Guo et al., 2018).

#### 4.1.2 2,4-Diaminoquinazoline-based inhibitors

**FIGURE 7:**
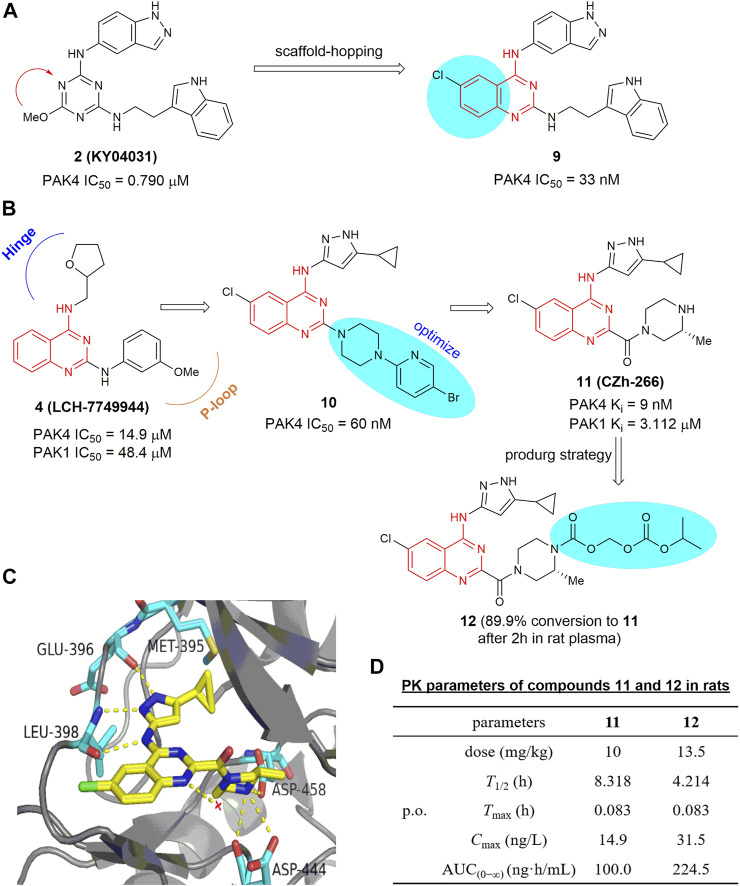
**(A)** Design of compound 9. **(B)** The optimization process leading to compounds 10 and 11. **(C)** The crystal structure of compound 11 in complex with PAK4 (PDB code: 5XVG). **(D)** PK parameters of compounds 11 and 12 in rats.

On the basis of analysis of the interaction mode of KY04031, an early developed modestly potent PAK4 inhibitor bearing a triazine core (IC_50_ = 0.790 μm) (Ryu et al., 2014), a scaffold-hopping approach was conducted recently by the Chen group (Hao et al., 2017), leading to a series of new PAK4 inhibitors containing a 2,4-diaminoquinazoline scaffold. Compound 9 (Figure 7A) exhibited a significant increased potency against PAK4 with an IC_50_ of 33 nm, likely due to the quinazoline core occupying the lipophilic phosphate-binding pocket. Compound 9 selectively inhibits PAK4 dependent proliferation of A549 cells and suppressed cell migration and invasion *via* regulation of the PAK4-LIMK1 signaling pathway. These results suggest that 2,4-diaminoquinazoline 9 is a PAK4 inhibitor with good selectivity and safety *in vitro*. 2,4-Diaminoquinazoline 10 (Figure 7B) is another potent PAK4 inhibitor reported by the same group, bearing an 3-amino-5-cyclopropyl-pyrazole fragment to interact with the hinge-binding pocket and showing an IC_50_ value of 60 nm (Wu et al., 2018). Further efforts on optimization of the cellular activity by modifying the C2-amino fragment as the P-loop interaction pocket yielded 4-aminoquinazoline-2-carboxamide 11 (Figure 7B), showing robust PAK4 potency with a K_i_ value of 9 nm and a remarkably high selectivity of 344-fold over PAK1 (K_i_ = 3.1 μm) (Hao et al., 2018). The crystal structure of PAK4 with 11 shows that the C4-amino pyrazole fragment forms three hydrogen bondings with Glu 396 and Leu 398 in the hinge region, and the cyclopropyl moiety forms hydrophobic interactions with Met 395 and Val 335 (Figure 7C). It is of note that the carboxylic side chain of Asp 444 in PAK4 forms electrostatic interactions with the NH in the C2-carbonylpiperazine motif (3.4 Å) by inducing a conformational alteration, whereas the side chain of Asp 407 in PAK1 is oriented away from this pocket, hence unable to form electrostatic interactions with compound 11. This might be a crucial element for the high potency and PAK4/PAK1 selectivity of compound 11. Compound 11 has promising drug-like properties and ADMET profile, however, high clearance and low plasma exposure were observed in rats. N-Isopropyl-oxy-carbonyloxymethyl 12 (Figure 7B) was then designed as a prodrug of 11 to block the metabolic hotspot (Guo et al., 2020). It was found that nearly 90% of prodrug 12 was transformed into the prototypic drug 11 after incubating with rat plasma for 2 h with over 2-fold increase in both C_max_ and AUC_(0−∞)_ after oral administration (Figure 7D). In HCT-116 and B16F10 xenograft models, oral administration of 12 at 50 mg/kg significantly suppressed the tumor growth with inhibition rates of 65% and 63%, respectively in the two mice models (Guo et al., 2020).

#### 4.1.3 Other bicyclic p21-activated kinase 4 inhibitors

**FIGURE 8:**
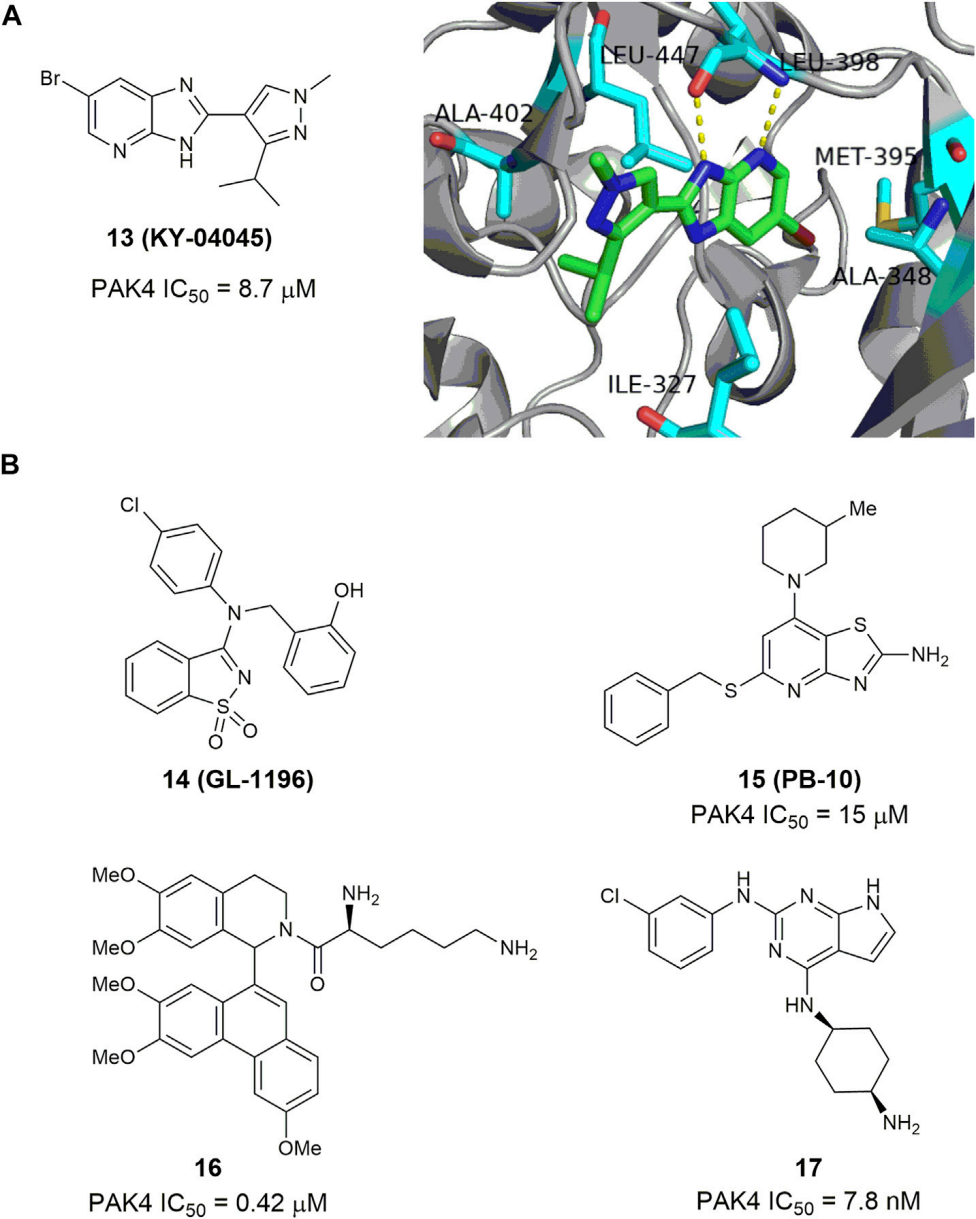
**(A)** The structure of compound 13 and its crystal structure in complex with PAK4 (PDB code: 5I0B). **(B)** The structure and activity of bicyclic PAK4 inhibitors 14–17.

Through site-directed fragment-based virtual screening, Park and co-worker reported an imidazo [4,5-b] pyridine-based PAK4 inhibitor 13 (KY-04045) (Park et al., 2016), showing a modest IC_50_ value of 8.7 μm. The co-crystal structure of PAK4 in complex with 13 shows that the imidazopyridine and pyrazole rings form sufficient hydrogen-bonding interactions in the hinge loop interaction (Figure 8A). 3-Aminobenzo [d] isothiazole 1,1-dioxide 14 (GL-1196) (Figure 8B) is another bicyclic PAK4 inhibitor discovered by screening of a series of small molecular compounds (Zhang et al., 2016). At concentrations of higher than 20 μm, this compound effectively suppressed phosphorylation of PAK4 and the proliferation of human gastric cancer cells through downregulation of both PAK4/c-Src/EGFR/cyclinD1 signaling and CDK4/6 expression (Zhang et al., 2016). Compound 15 (Figure 8B) bearing a thiazole [4,5-d] pyrimidine scaffold was also reported to have modest potency against PAK4 with an IC_50_ value of 15 μm (Li Z et al., 2020). It showed moderate potency against proliferation of HTC-116 cells with an IC_50_ value of 4.3 μm (Li R et al., 2020), and reduced cell migration and invasion by inhibiting the PAK4/LIMK1/Cofilin signaling pathway (Li Z et al., 2020). Phenanthryl-tetrahydroisoquinoline 16 (Figure 8B) is a moderately potent PAK4 inhibitor with an IC_50_ value of 420 nm (Hao et al., 2016). In the A549 and MCF-7 cancer cells, this compound showed good cell grow inhibition with IC_50_ values of 0.28 and 0.83 μm, respectively. Immunoprecipitation assay revealed that compound 16 bonded with both N- and C- terminals of PAK4. In the xenograft model derived from breast cancer A549 cells, oral administration of 16 at a dose of 50 mg/kg showed a growth inhibitory rate over 50% with no apparent toxicity. Recently, compound 17 (Figure 8B) bearing a 7H-pyrrolo [2,3-d] pyrimidine scaffold was reported to display excellent activity against PAK4 and proliferation of MV4-11 cells with IC_50_ values of 2.7 and 7.8 nm, respectively (Wang et al., 2022). It inhibits phosphorylation of PAK4 dose-dependently, induces cell apoptosis and arrests MV4-11 cells at G0/G1 phase. Molecular docking analysis showed that the pyrrolo [2,3-d] pyrimidine core might bind to the ATP-binding cleft of PAK4 through various hydrogen bonding network.

#### 4.1.4 Monocyclic p21-activated kinase 4 inhibitors

**FIGURE 9:**
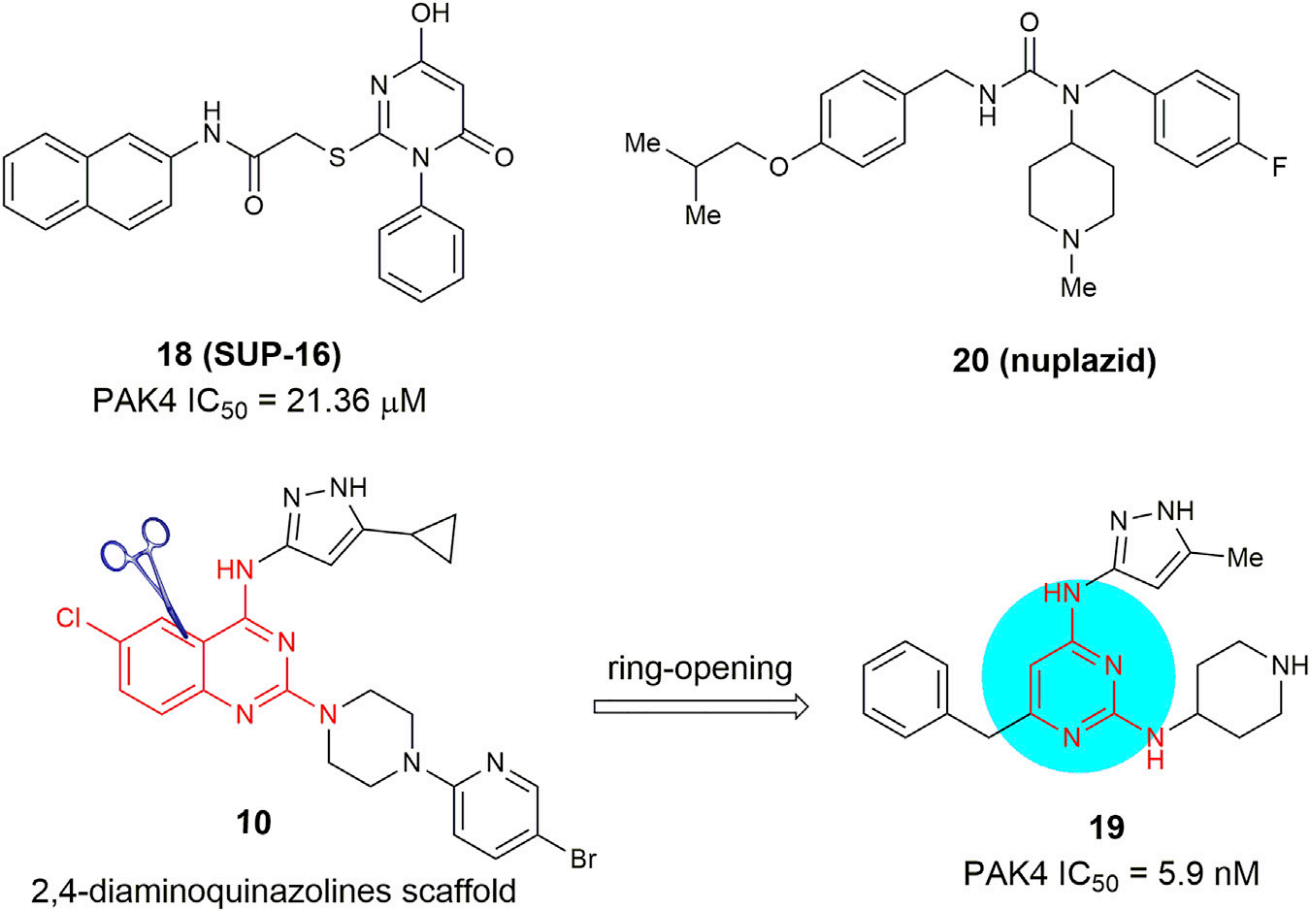
Monocyclic PAK4 inhibitors 18–20.

Through structure-based virtual screening, 6-hydroxy-2-mercapto-3-phenylpyrimidin-4(3H)-one 18 (SUP-106, Figure 9) was identified as a novel monocyclic PAK4 inhibitor (Song et al., 2019). This compound binds to the C-terminus of the PAK4 kinase domain with an IC_50_ value of 21.36 μm and has no significant binding to PAK1, PAK5, and PAK6. It suppressed the invasion of human gastric cancer SGC7901 cells and alleviated metastasis related SCG10 phosphorylation through downregulation of the PAK4/LIMK1/cofilin and the downstream protein SCG10 signaling pathways. Meanwhile, Cheng and co-workers (Qin et al., 2020) recently reported a monocyclic PAK4 inhibitor 19, bearing a 2,4-diaminopyrimidine scaffold. This compound can be viewed as a ring-opening analogue of their early reported 2,4-diaminoquinazolines (e.g., 10). This compound retains high potency against PAK4 with an IC_50_ of 5.9 nm but displays much lower anti-proliferative activity against A549 cells with an IC_50_ value of 2.5 μm. Very recently, Liu and co-workers (Wei et al., 2021) conducted a drug repurposing screening on a library of the FDA-approved drugs and identified that the anti-Parkinson drug Nuplazid (compound 20), a 5-HT_2 A_ receptor inverse agonist, suppressed the growth of several esophageal squamous cell carcinoma (ESCC) cells with IC_50_ values of approximately 10 µm. Mechanism studies confirmed that this compound is an ATP competitive inhibitor and interacts with Asp 444 in the PAK4 kinase domain, Moreover, compound 20 significantly suppressed the growth of ESCC PDX model, thus is worthy of further investigation as an effective PAK4 inhibitor.

#### 4.1.4 Other p21-activated kinase 4 inhibitors

PAKib is a new PAK4 inhibitor reported very recently with 500 nm of IC_50_ against PAK4 (He et al., 2022). PAKib inhibits the proliferation of pancreatic cancer cell lines derived from human or murine by inducing G2/M phase arrest (He et al., 2022). In the murine pancreatic cancer model, PAKib suppresses tumor growth with a dose of 40 mg/kg. The tumor volume reduces 47% after treated PAKib for 20 days compared to the control group (He et al., 2022). Moreover, the combination of PAKib and gemcitabine, the tumor volume is further decreased compared to the single-agent group (He et al., 2022). These characteristics demonstrated that PAKib is a potent PAK4 inhibitor. However, the structure has not been revealed.

In addition, many natural products have also been reported as potential PAK4 inhibitors, including vitexin, emodin, ziganein, and kaempferol (Arowosegbe et al., 2020; Iwaloye et al., 2021). However, these compounds generally possess much modest potency against PAK4, and the underlying mechanism needs to be further validated as well.

### 4.2 Allosteric inhibitors

#### 4.2.1 KPT-9274 (ATG-019) and analogues

**FIGURE 10:**
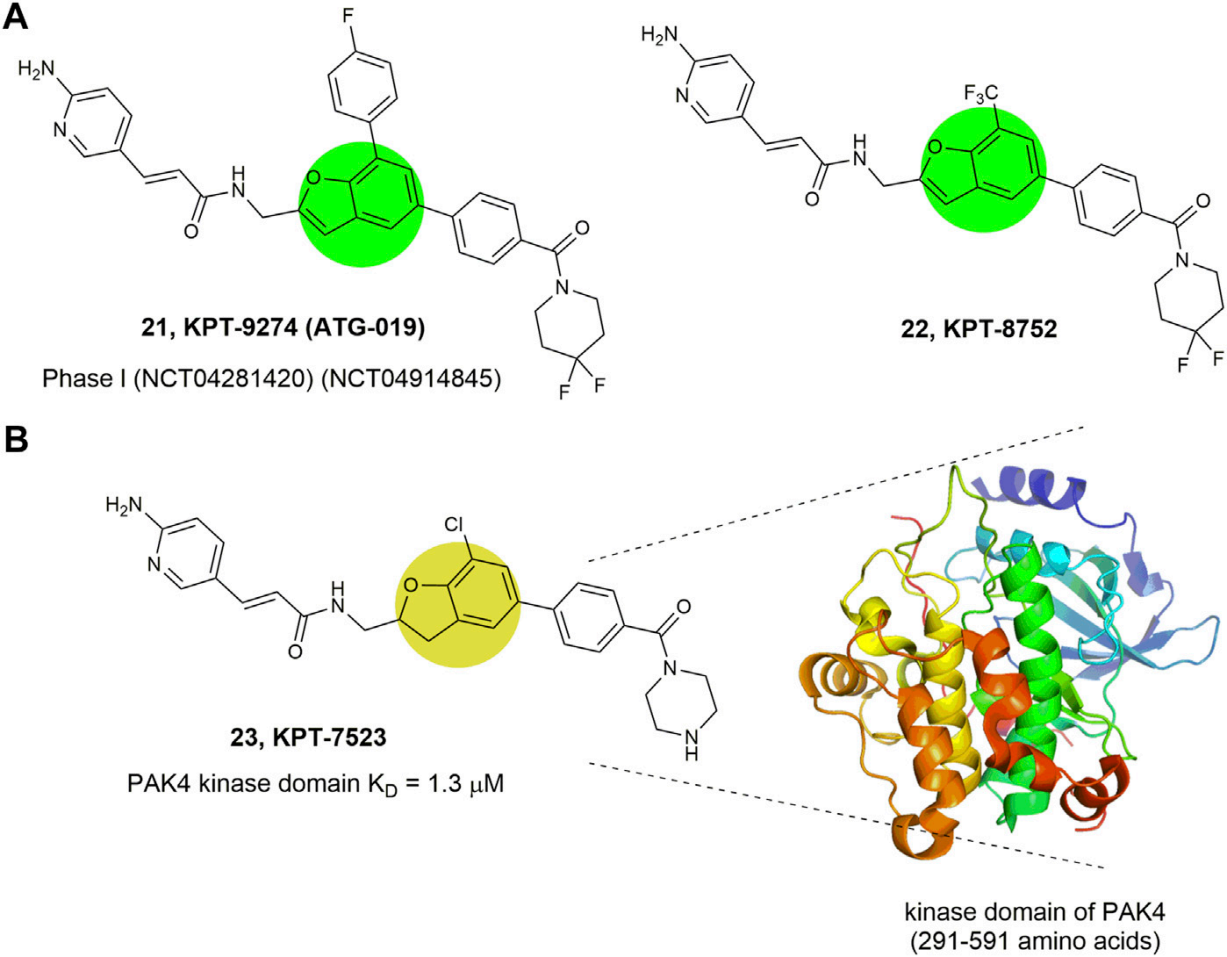
Structure of PAK4 allosteric inhibitors. **(A)** 21 and 22 bearing a benzofuran core. **(B)** 23 bearing a 2,3-diphydrobenzofuran core and its binding mode with the kinase domain of PAK4. The activity (IC_50_) of these inhibitors against PAK4 kinase domain was not reported.

Compounds 21–23 (Figure 10) represent a series of novel PAK4 inhibitors developed by Karyopharm Therapeutics bearing a trisubstituted benzofuran or a 2,3-diphydrobenzofuran scaffold. Among them, KPT-9274 (compound 21) is an orally bioavailable PAK4 and NAMPT (nicotinamide phosphoribosyl transferase) dual targeted inhibitor with IC_50_ values of 120 nm against NAMPT, and currently being tested in phase Ⅰ clinic trial to treat solid tumors and non-Hodgkin’slymphoma (Abu Aboud et al., 2016). Mechanism study reveals that KPT-9274 downregulates the expression of PAK4 and its downstream proteins without direct binding with the ATP pocket of PAK4, therefore referring as a PAK4 allosteric modulator (PAM). To illustrate the allosteric binding mechanism, the regulatory domain (1–290 amino acids) and kinase domain (291–591 amino acids) of PAK4 were prepared and KPT-7523 (compound 23), a close analogue of KPT-9274 was used as a probe. It was found that although KPT-7523 has a Michael acceptor, but no covalent binding was identified. Instead, KPT-7523 was found specifically binding to the kinase domain of PAK4 with a favorable K_D_ of 1.3 μm (Fulciniti et al., 2017). These results might suggest a similar binding mode for KPT-9274, which binds to the PAK4 kinase domain, rather than the ATP pocket.

## 5 Preclinical and clinical studies of p21-activated kinase 4 inhibitors

### 5.1 PF-3758309

Among all the reported potent PAK4 inhibitors (Table 1), PF-3758309 developed by Pfizer is the first PAK4 inhibitor approaching into clinical trials. Although it was designed for PAK4, high potency was observed for other PAK isoforms (e.g., PAK1 K_i_ = 13.7 ± 1.8 nm, PAK4 K_i_ = 18.7 ± 6.6 nm) (Murray et al., 2010). PF-3758309 exhibited excellent anti-proliferation activity against lung, pancreatic, breast and colon cancer cell lines with IC_50_ values less than 10 nm. Especially for HCT116 by showing an IC_50_ value of 0.24 nm (Murray et al., 2010; Pitts et al., 2013). In HCT-116 xenograft model, oral administration of PF-3758309 at 7.5, 15, and 20 mg/kg significantly suppressed the tumor growth with inhibition rates (TGI) of 64%, 79%, and 97%, respectively (Murray et al., 2010). Recent study shows that PF-3758309 suppresses adult T-cell leukemia (ATL) in xenograft model with TGI of 87% at a daily dose of 12 mg/kg (Chung et al., 2019). Furthermore, in PANC-02 orthotopic cancer model, treatment with PF-3758309 increased the levels of CD3^+^ and CD8^+^ T cells in tumor tissue (Wang et al., 2018), indicating that this compound induces immune infiltration. Taken together, the preclinical studies proved that PF-3758309 is a potent druglike PAK4 inhibitor suitable for clinical trials for treating colon, leukemia, and pancreatic cancers with potentials to improve immune microenvironment.

**TABLE 1:** Summary of the PAK4 inhibitors.

Compound	Inhibition activity (IC_50_)	Cocrystal (PDB code)	Ref	Compound	Inhibition activity (IC_50_)	Cocrystal (PDB code)	Ref
1 (PF-03758309)	19 nm	2X4Z	Murray et al. (2010)	16 (12a^e^)	420 nm	—	Hao et al. (2016)
2 (KY-04031)	790 nm	4NJD	Ryu et al. (2014)	17 (5n^e^)	2.7 nm	—	Wang et al. (2022)
3 (GNE-2861)	7.5 nm	4O0V	Staben et al. (2014)	18 (SUP-106)	21.36 μm	—	Song et al. (2019)
4 (LCH-7749944)	14.9 μm	—	Zhang et al. (2012)	19 (B6^e^)	5.9 nm	—	Qin et al. (2020)
5 (CGP74514)	—	2CDZ	Eswaran et al. (2007)	20 (Nuplazid)	—	—	Wei et al. (2021)
6 (19^e^)	K_i_ = 4 nm	—	Rudolph et al. (2015)	21 (KPT-9274)	—	—	Fulciniti et al. (2017)
7 (12g^e^)	27 nm	—	Guo et al. (2017)	22 (KPT-8752)	—	—	Fulciniti et al. (2017)
8 (10a^e^)	25 nm	—	Guo et al. (2018)	23 (KPT-7523)	—	—	Fulciniti et al. (2017)
9 (9d^e^)	33 nm	—	Hao et al. (2017)	29^e^	K_i_ = 455 nm	5BMS	Crawford et al. (2015)
10 (8d^e^)	60 nm	—	Wu et al. (2018)	3^e^	K_i_ = 64 nm	4APP	Guo et al. (2012)
11 (CZh-226)	K_i_ = 9 nm	5XVG	Hao et al. (2018)	8^e^	K_i_ = 68 nm	4O0X	Staben et al. (2014)
13 (KY-04045)	8.7 μm	5I0B	Park et al. (2016)	30^e^	K_i_ = 51 nm	5XVA	Hao et al. (2018)
14 (GL-1196)	—	—	Zhang et al. (2016)	10a^e^	K_i_ = 710 nm	5XVF	Hao et al. (2018)
15 (PB-10)	15.12 μm	—	Li R et al. (2020)	PAKib^e^	500 nm		He et al. (2022)

e corresponding compound numbers in the literatures.

Unfortunately, clinical treatment with PF-3758309 showed no objective response (Rudolph et al., 2015). In dogs and rats, this compound has oral bioavailability of 39%–76% and 20%, respectively, however, the corresponding values was only approximately 1% in human (Rudolph et al., 2015). In addition, certain adverse events were observed in tumor patients, including neutropenia and gastrointestinal side effects. These unexpected adverse effects prevented further clinical investigation of PF-3758309 and subsequent phase I clinical trials were terminated (Table 2).

**TABLE 2:** Progress in clinical trials of PAK4 inhibitors.

Compound	Clinical trials	Disease	Status
PF-03758309	NCT00932126 (phase Ⅰ)	Solid tumors	Terminated
KPT-9274	NCT02702492 (phase Ⅰ)	Solid tumors	Terminated
	NCT04281420 (phase Ⅰ)	Solid tumors, non-hodgkin’s lymphoma	Recruiting
	NCT04914845 (phase Ⅰ)	Relapsed and refractory acute myeloid leukemia	Recruiting

### 5.2 KPT-9274

Pancreatic carcinoma (PCC) is one of the most malignant tumors with high lethality worldwide and its drug treatment remains an unmet medical need (Siegel et al., 2022). Recent studies reported that PAK4 is overexpressed in various PCC cells and mediates the proliferation of these cells (Thillai et al., 2018). Therefore, PAK4 has been proposed a pivotal target for pancreatic carcinoma. KPT-9274, as a selective and allosteric PAK4 inhibitor, significantly inhibits the proliferation of pancreatic cancer cells with preference over normal human ductal epithelial (Aboukameel et al., 2017). In the pancreatic cancer stem-like cells (CSCs), which are resistant to traditional chemotherapy, treatment with KPT-9274 suppressed the formation of spheroids and reduced the epithelial-mesenchymal transition markers (CD44^+^, EpCAM^+^) (Aboukameel et al., 2017). In BON-1 PCC xenograft, treatment with KPT-9274 at 150 mg/kg QD significantly suppressed tumor growth with two out of five tumors completely regressed (Mpilla et al., 2019). In addition, since the BON-1 cell is inherently resistant to the mTOR inhibitor Everolimus, a synergic lethality of the combination of the two drugs was anticipated. *In vitro*, KPT-9274 was found to synergize with Everolimus in cell growth inhibition, colony suppression, and glucose uptake. *In vivo*, the combination of KPT-9274 (150 mg/kg) with Everolimus (2.5 mg/kg) produced a statistically significant suppression in tumor growth, a scenario not observed for single agent of Everolimus (Mpilla et al., 2021). Mechanism study reveals that resistance to Everolimus is mainly due to activation of mTORC2, and treatment with the combination efficaciously resensitizes to BON-1 cell lines (Mpilla et al., 2021).

In the meantime, PAKs also play important roles in leukemic stem cells and several resistant cancer cells (Wu and Jiang, 2022). KPT-9274 was found to effectively suppress the cell proliferation of multiple myeloma (Fulciniti et al., 2017), leukemia (Mitchell et al., 2019), and lymphoma (Khan et al., 2021) in a time- or dose-dependent manner with no damage on the viability of normal cells such as bone marrow stromal cells (Fulciniti et al., 2017), normal hematopoietic progenitors (Mitchell et al., 2019), and peripheral blood mononuclear cells (Khan et al., 2021). Mechanism studies reveal that treatment with KPT-9274 induces apoptosis and cell cycle arrest, evidenced by the elevation of apoptosis-related markers cleaved caspase 3 and cleaved PARP in REH and SUP-B15 cell lines (Takao et al., 2018), and reduction of cell cycle markers (Khan et al., 2021) CDK2 and CDK4. Meanwhile, as a NAMPT inhibitor, KPT-9274 disrupts the binding of nicotinamide with NAMPT (Mitchell et al., 2019), thus decreasing the NAD/NADH ratios and the levels of ATP in both WSU-DLCL2 and WSU-FSCCL cells (Khan et al., 2021). Furthermore, a recent report reveals that KTP-9274 disrupts lipid homeostasis and induces leukemic stem cells apoptosis by targeting NAMPT (Subedi et al., 2021). In MM1S and OPM2 murine models, exposure to KPT-9274 (100 mg/kg) significantly suppressed tumor growth (Fulciniti et al., 2017). Similar tumor suppression effects were observed as well in the MV4-11 acute myeloid leukemia (AML), B-cell acute lymphoblastic leukemia (ALL) and Non-Hodgkin’s lymphomas (NHL) xenograft (Takao et al., 2018; Mitchell et al., 2019; Khan et al., 2021). More appealingly, in the WSU-FSCCL xenograft model (Khan et al., 2021), systemic KPT-9274 treatment remarkably prolonged the life span of mice with 50% of cure, in parallel with reduction in both total and phosphorylated PAK4 as well as increased pro-apoptotic cascade.

In addition, more combinations of KPT-9274 with drugs of various mechanisms were investigated and synergetic effects were generally achieved. For example, administration of KPT-9274 with the DNA-damage agent bendamustine afforded a synergetic antitumor effect higher than each drug in the Waldenstrom macroglobulinemia (WM) BCWM-1 xenograft model (Li et al., 2019). In the MOLM13 xenograft model, combination of KPT-9274 with the HDAC inhibitor AR-42 showed a much prolonged life span than each monotherapy (41 vs. 27 or 29 days) (Zhang et al., 2021).

Recently, KPT-9274 was also reported to have therapeutical potential for triple negative breast cancer (TNBC). PAK4 is overexpressed in TNBC cells and the levels of PAK4, phosphorylated PAK4 and downstream phosphorylated β-catenin as well as phosphorylated cofilin were remarkedly reduced after treatment with KPT-9274 for 72 h in SUM159 TNBC cells (Rane et al., 2017). Among the tested three TNBC cell lines (MDA-MB-231, MDA-MB-468, SUM159), MDA-MB-468 and SUM159 cells were particularly sensitive, and their growth was completely inhibited after treatment with KPT-9274 (300 nm) (Rane et al., 2017). Mechanism study indicates that the antiproliferative effect of KPT-9274 in TNBC cells is likely due to its inhibition of Rictor, a component of mTORC2, thus resulting in regulation of cell growth and metabolism (Cordover et al., 2020). In the three xenograft TNBC models, oral administration of KPT-9274 (150 mg/kg or 100 mg/kg) significantly suppressed tumor growth (Rane et al., 2017).

Moreover, application of PAK4 inhibitors in renal cell carcinoma (RCC) has been reported as well (Abu Aboud et al., 2016). Treatment of KPT-9274 attenuated the viability of RCC (Caki-1 and 786-O) cells and induced G_2_/M arrest and apoptosis. In the meantime, c-Myc and Cyclin D1, the two RCC oncogenic proteins, were decreased drastically, and the NAD levels were also reduced due to its inhibition of NAMPT (Abu Aboud et al., 2016). Further, in the 786-O xenograft model, tumor growth was significantly suppressed, after treatment of KPT-9274 (100 mg/kg) (Abu Aboud et al., 2016).

Since previous studies have revealed that PAK4 is involved in tumor cell infiltration by immune cells through regulation of WNT/β-catenin pathway, PAK4-targeted inhibitors may have synergic effect with PD-1/PD-L1 checkpoint blockades. Ribas and co-workers (Abril-Rodriguez et al., 2020) recently conducted a transcriptional analysis of tumor biopsies, and found that PAK4 expression is enriched in immune cell poorly infiltrated non responding tumors. In the PD-1 blockade insensitive B16 murine melanoma model, the combination of KPT-9274 with an anti-PD-1 agent showed remarkable suppression of tumor growth compared with anti-PD-1 agent or KPT-9274 alone (*p* = 0.0007 vs. 0.01) (Abril-Rodriguez et al., 2020). Meanwhile, inhibition of PAK4 can normalize the tumor vascular microenvironment and sensitize glioblastoma (GBM) to chimeric antigen receptor–T (CAR-T) cell immunotherapy. In the mouse GL261 GBM model, monotherapy of either KPT-9274 or CAR-T does not suppress the growth of tumors significantly, however, the combination of both exerts a nearly 80% tumor growth with much prolonged survival (Ma et al., 2021). Taken together, PAK4 is an appealing immunotherapeutic target and PAK4 inhibitors provide a unique prospect to combat tumors with immune poorly inflamed and resistant to checkpoint therapies.

At present, there are three phase Ⅰ clinical trials of KPT-9274 in progress for solid tumors, Non-Hodgkin’s lymphoma, and acute myeloid leukemia (NCT02702492, NCT04281420, NCT04914845), respectively. Among them, “NCT02702492” was terminated in 2021, but the detailed results have not been disclosed yet. In addition, the other two clinical trials are still in the process of patient recruiting.

## 6 Conclusion and perspectives

Since the first recognition in 1994, the PAK family has gained a continuously increasing interest both in the biological function and their roles in disease modulation. Culminating evidences have shown that the aberrant PAK4 signaling is critically implicated in nearly all hallmarks of cancer and overexpression of its mRNA or proteins has been observed in a large number of diverse human tumors, including those highly malignant ones. Genetic knockout or pharmacological inhibition have postulated that PAK4 is an extremely attractive drug target and many PAK4 inhibitors either as ATP competitive or as allosteric inhibitors bearing multiple chemotypes have been developed and tested as a first line or as adjuvant cancer treatment. Unfortunately, the majority of current reported PAK4 inhibitors suffer from poor selectivity, insufficient antitumor efficacy, and unfavorable drug-like properties. Therefore, by far, there are only two PAK4 inhibitors (PF-3758309, KPT-9274) entering into clinical trials. PF-3758309 is the first PAK inhibitor tested in the clinical trial, but was terminated due to its poor PAK4 selectively and adverse events together with pharmacokinetic issues observed from patients during phase I study. The emergence of the allosteric inhibitor KPT-9274 draws a new prospect for PAK4 inhibitors. KPT-9274 not only has high selectivity for PAK4 with an allosteric mechanism and also is a potent inhibitor targeting NAMPT, an enzyme important for tumor development and progression as well. In addition, PAK4 was found to play an active role in excluding tumor-specific T cells from the tumor microenvironment, and the PAK4 inhibitor KPT-9274 has showed synergic effects to increase tumor-specific T cell infiltration and hence sensitize non-responsive or resistant tumors to PD-1 blockade therapy. Therefore, KPT-9274 has great potentials either as monotherapy or as combinations with immunotherapy to treat both immune inflamed and poorly inflamed/resistant tumors. However, the fate of KPT-9274 and other PAK4 inhibitors has to wait for the overall outcomes of the final clinical trials on human patients.

In addition, it has to be alert that although the mRNA and protein levels of PAK4 are generally observed in cancer cells, the precise mechanism underlying this increase and involvement of PAK4 in the proliferation and transformation of cancer cells are not completely understood. Some studies show that modulation of mTOR and the subsequent WNT/β-catenin pathway might be a critical mechanism for PAK4 in cell proliferative, metabolism, and immune cell infiltration, however, PAK4 certainly has many other functions and many other pathways can be involved in the WNT/β-catenin signaling. Therefore, much effort is need to further investigate the fundamental biological mechanisms PAK4 underlying, which will be critically important for patient stratification and biomarker selectivity for clinical trials.
